# Supplementary material for: Modeling early stage atherosclerosis in a primary human vascular microphysiological system
Source: Nat Commun. 2020 Oct 27;11:5426. doi: 10.1038/s41467-020-19197-8 (PMC7591486; doi:10.1038/s41467-020-19197-8)
Supplement: Supplementary file 1 — Supplementary Information [file 41467_2020_19197_MOESM1_ESM.pdf]

Supplementary Information

Modeling Early Stage Atherosclerosis in a Primary Human Vascular Microphysiological System

Supplementary Figures

Supplementary Figure 1……………………………………………………………………….2
Supplementary Figure 2……………………………………………………………………….3
Supplementary Figure 3……………………………………………………………………….4
Supplementary Figure 4……………………………………………………………………….5
Supplementary Figure 5……………………………………………………………………….6
Supplementary Figure 6……………………………………………………………………….7
Supplementary Figure 7……………………………………………………………………….8
Supplementary Figure 8……………………………………………………………………….9
Supplementary Figure 9……………………………………………………………………….10
Supplementary Figure 10……………………………………………………………………...11
Supplementary Figure 11……………………………………………………………………..12

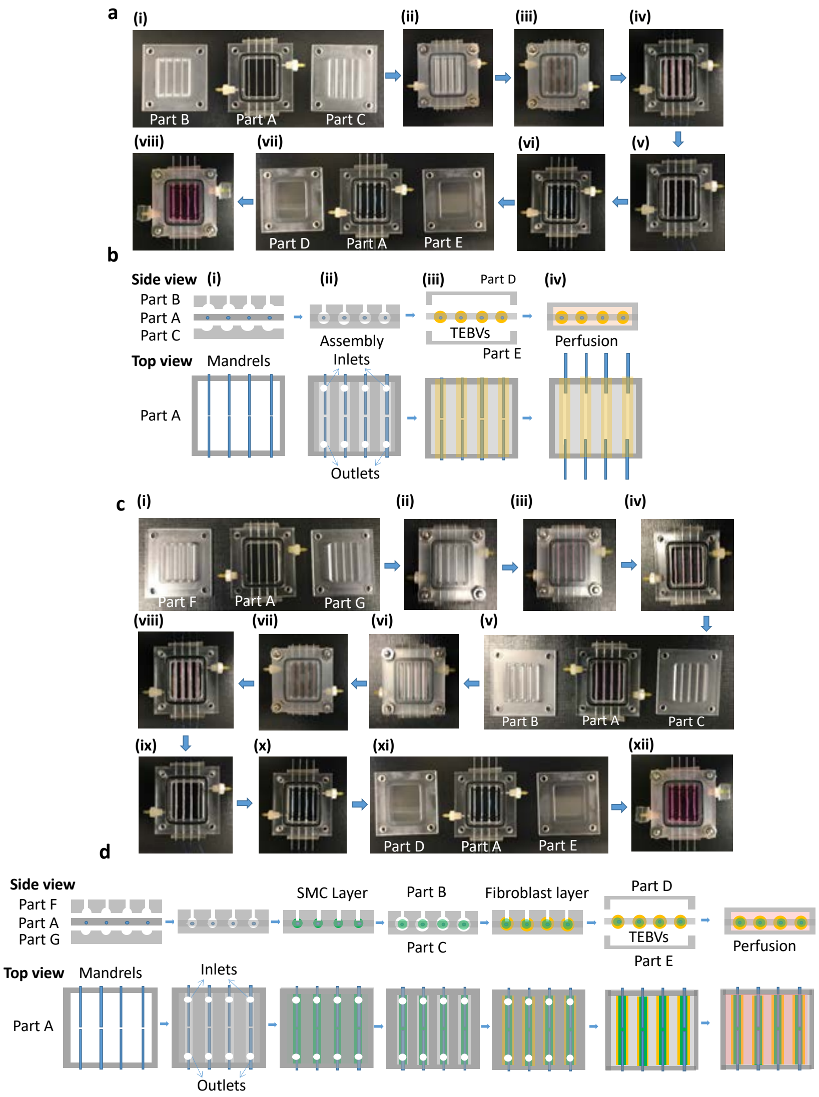

Figure S1. TEBV fabrication. a. Steps in 2-layer TEBV fabrication: (i) the parts to assemble seeding mold; (ii) the assembled seeding chamber; (iii) loading the collagen/fibroblast mixture into molds; (iv) removal of Parts B and C; (v) dehydration of TEBVs; (vi) drawing out mandrels and suture of TEBVs to separated mandrel to link TEBV to flow circuit; (vii) the parts for the perfusion chamber; (viii) the assembled perfusion chamber. **b.** Schematic diagrams of side and top views of 2-layer TEBV fabrication chamber. **c.** Steps of 3-layer TEBVs fabrication (i) the parts to assemble seeding mold of middle layer; (ii) the assembled seeding chamber of middle layer; (iii) loading the collagen/SMC mixture into the molds; (iv) removal of Parts F and G; (v) parts to assemble seeding mold of outer layer; (vi) the assembled seeding chamber of outer layer; (vii) loading the collagen/fibroblast mixture into the molds; (viii) removal of Parts B and C; (ix) dehydration of TEBVs; (x) removing the mandrels and suture of TEBVs to separated mandrel to link TEBV to flow circuit; (xi) the parts for the perfusion chamber; (xii) the assembled perfusion chamber. **d.** Schematic diagrams of side and top views of 3-layer TEBV fabrication.

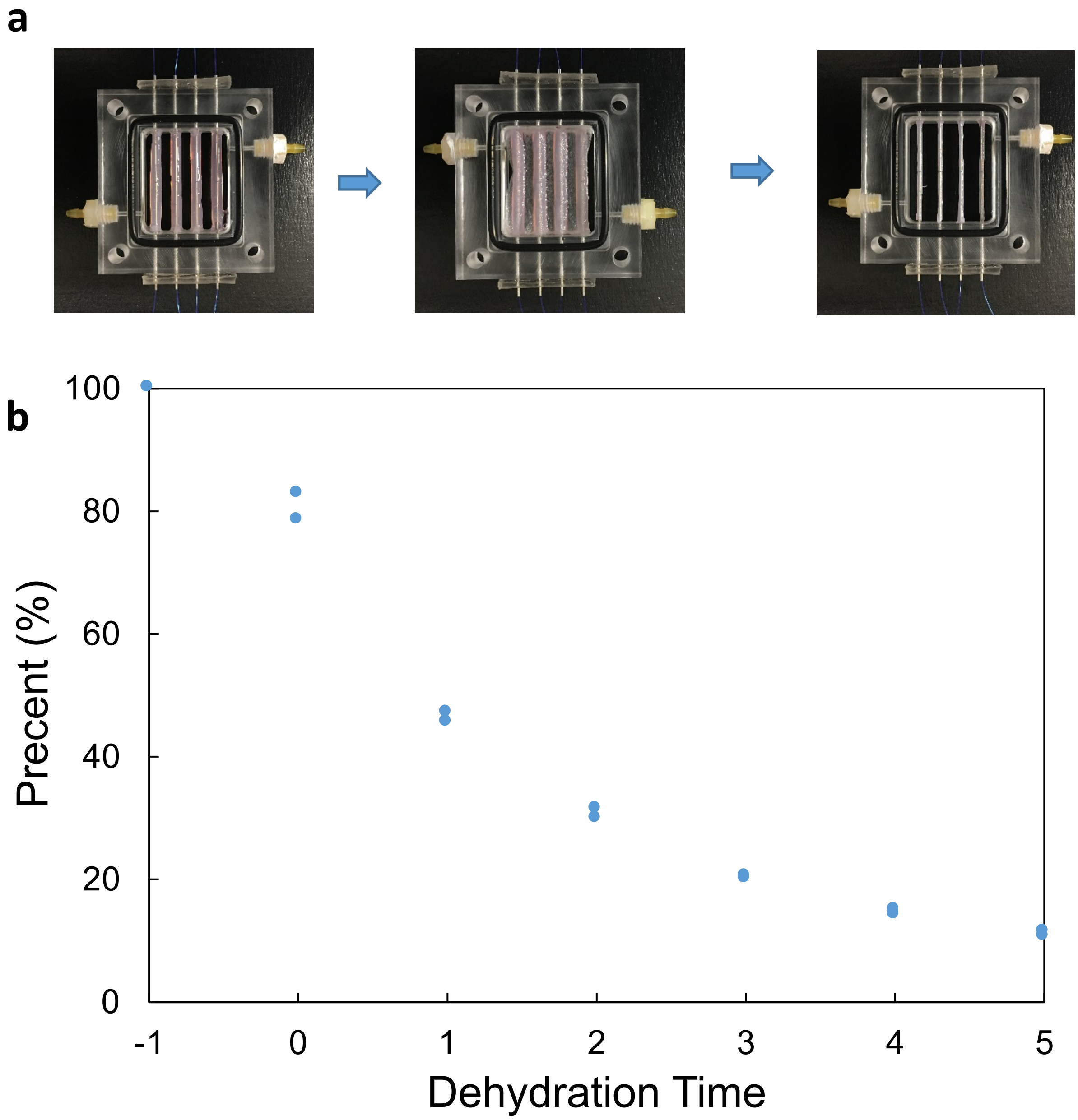

Figure S2. Plastic compression to remove water and form dense collagen gel TEBVs.a. dehydration of TEBVs with Kimwipes. **b.** Percent change of TEBV weight with dehydration time. On the X-axis, -1 is the initial weight of collagen mixture injected into the mold). 0 is weight of TEBVs after peeling off the top and bottom layers. (mean±S.D., n=2 chambers). Subsequent numbers represent the number of times Kimwipes were applied to TEBVs.

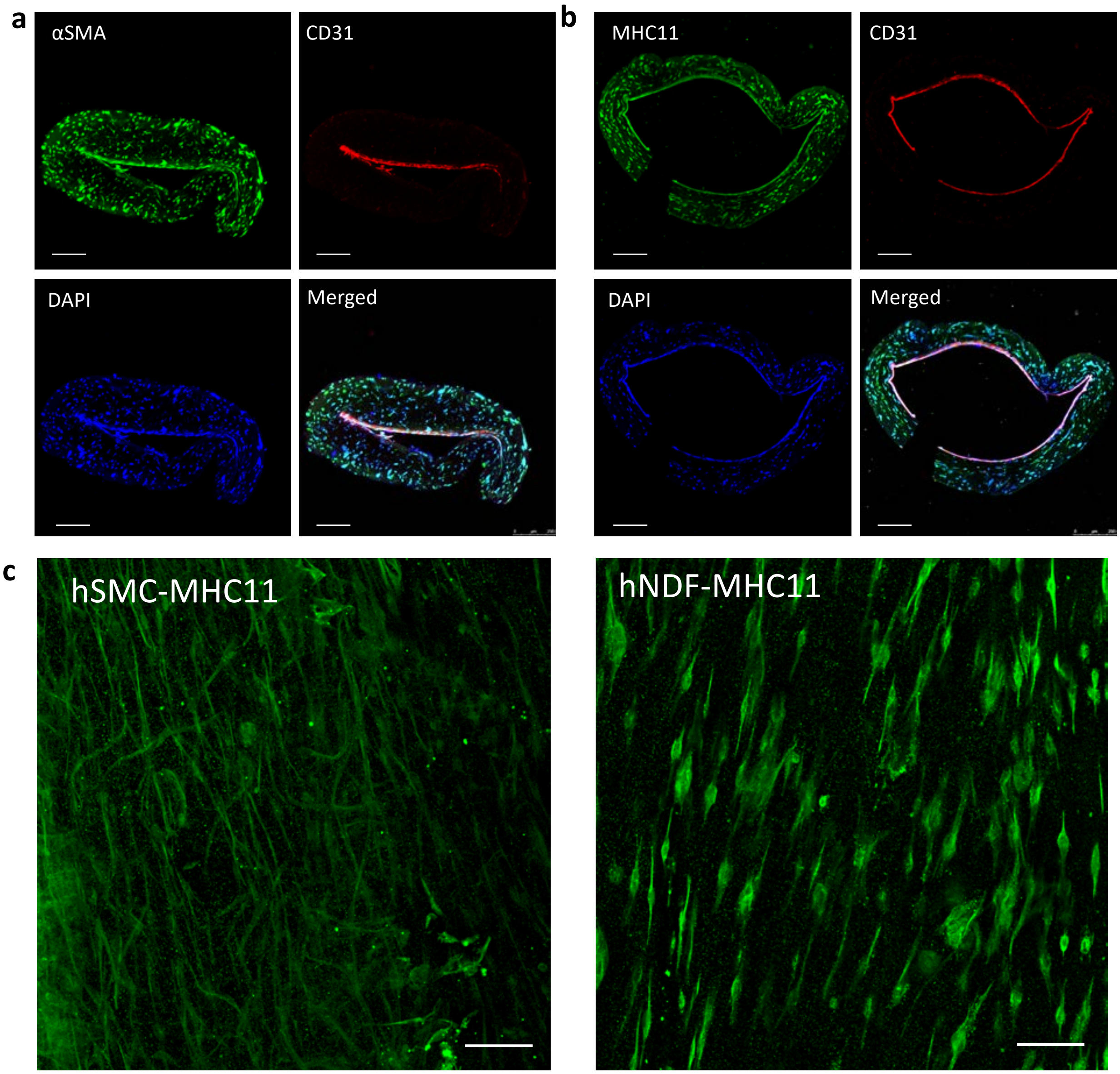

Figure S3. α smooth muscle actin (αSMA) (**a**) and myosin heavy chain 11 (MHC11) (**b**) are present in the hSMC and hNDF layers of 3-layer TEBVs (scale bar 200 μm). **(c)** Longitudinal view of MHC11 staining of hSMCs and hNDFs in 3-layer model (scale bar 100 μm).

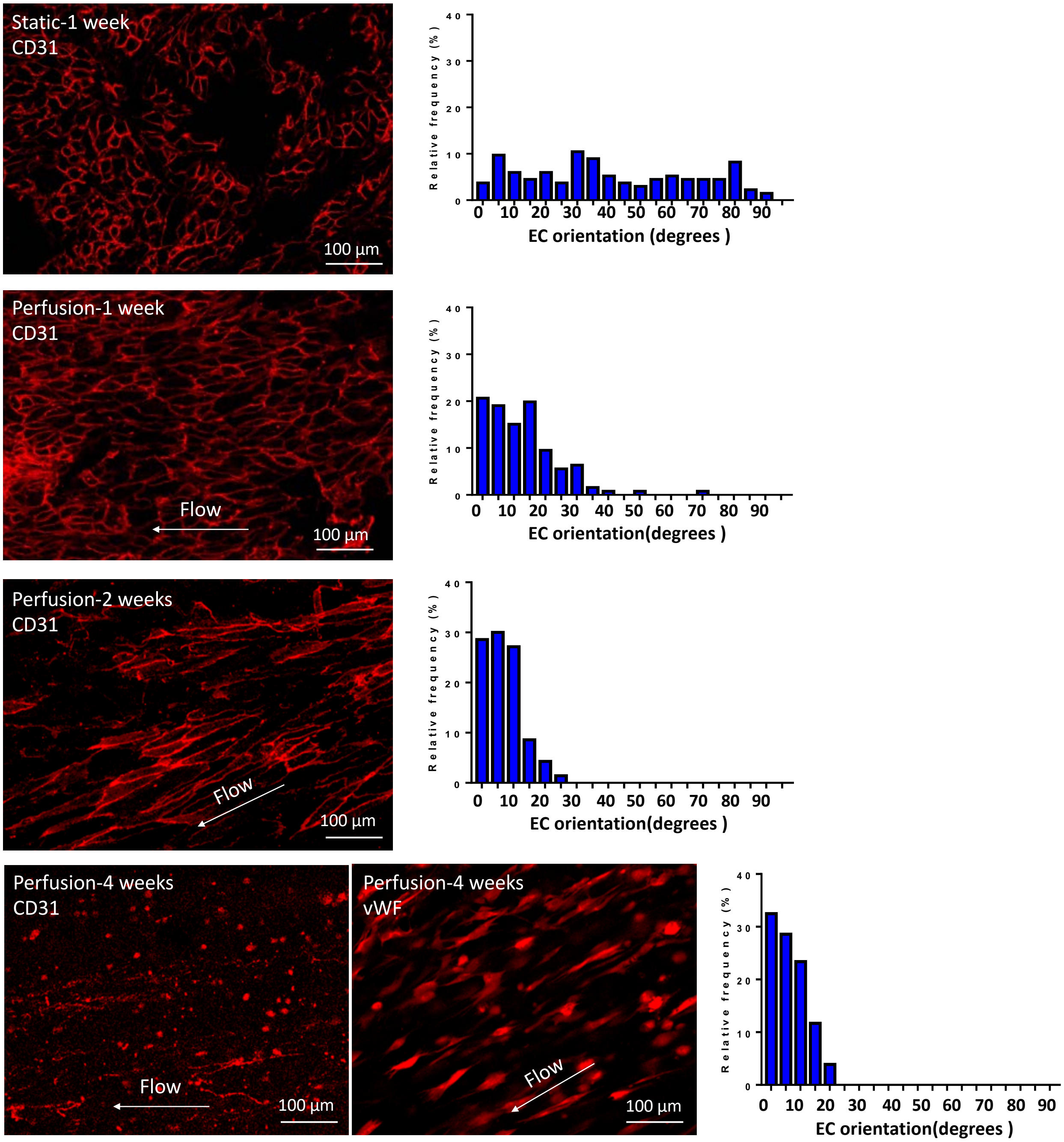

Figure S4. Characterization of endothelial cells in TEBVs following 4 weeks of perfusion. The CD31 staining indicated cell-cell contacts of ECs present during 2 weeks of perfusion. By 4 weeks of perfusion, CD31 was still present, but junctions were discontinuous. The analysis of ECs angles to flow displayed the alignment of ECs relative to the direction of flow. For static experiments, orientation was relative to horizontal.

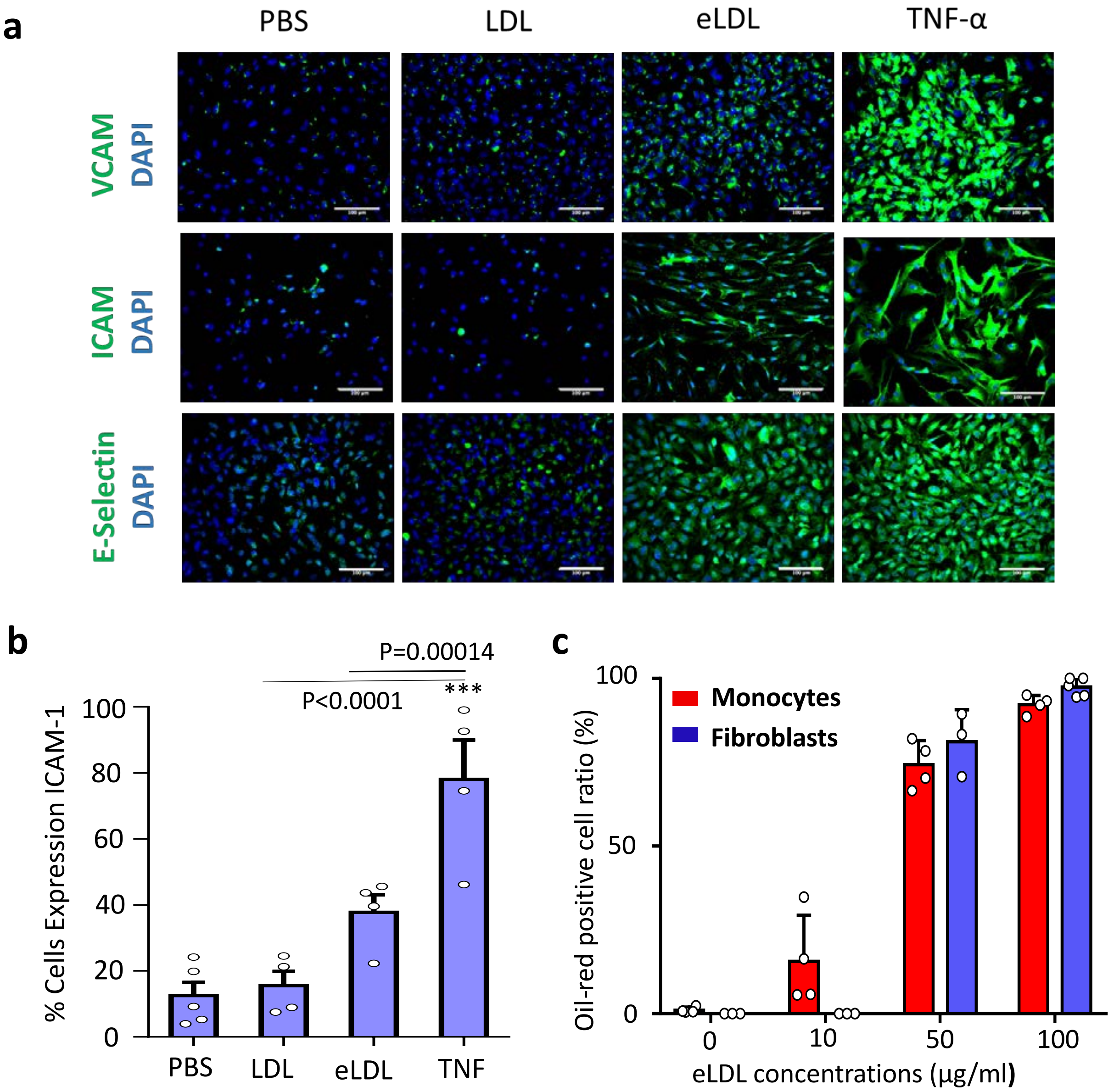

Figure S5. a. Expression of VCAM-1, ICAM-1 and E-selectin in endothelial cells after exposure to 100 µg/mL LDL or eLDL for 24 h or 100 U/mL TNFa for 4.5 h (scale bar: 100 µm). **b.** Quantification of cells expressing ICAM-1 (N = 5,4,4,4 independent experiments accordingly; Mean ± SEM., ***P<0.0001 compared to PBS group by one-way ANOVA and a Tukey post hoc test). **c.** Foam cells produced after exposure of hNDFs or primary macrophages to eLDL with various concentrations for 24 h (mean ± S.D., n=4 independent wells for monocytes and n=3,5 independent wells for fibroblasts). Fibroblasts exposed to 10 µg/ml eLDL for 24 h did not exhibit any Oil-red O staining.

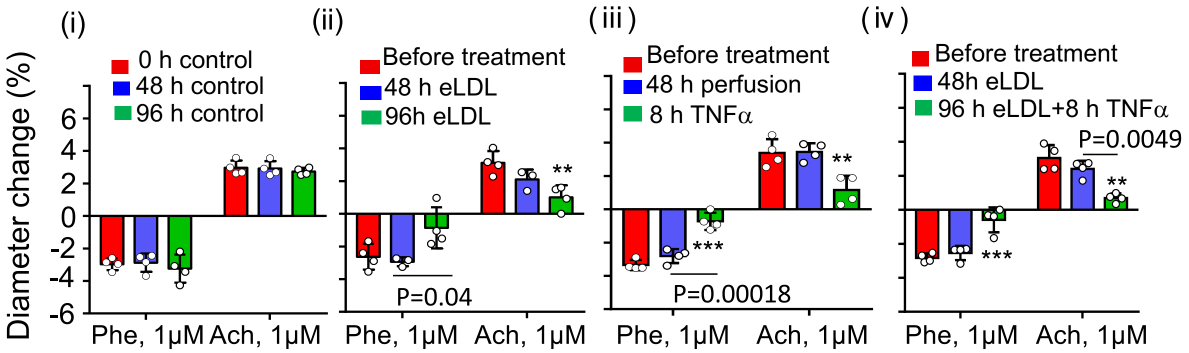

Figure S6. Vasoactivity of 3-layer TEBVs for control (i), 50 μg/ml eLDL (ii), 50 U/ml TNFα (iii), 96 h 50 μg/ml eLDL+8 h 50 U/ml TNFα (iv) (mean ± S.D., n=4 TEBVs, *P < 0.05, ** P < 0.01, ***P < 0.001 compared to before treatment). compared to before treatment group by one-way ANOVA and a Tukey post hoc test (ii) ** P=0.0070; (iii) ***P<0.0001, **P=0.0058.

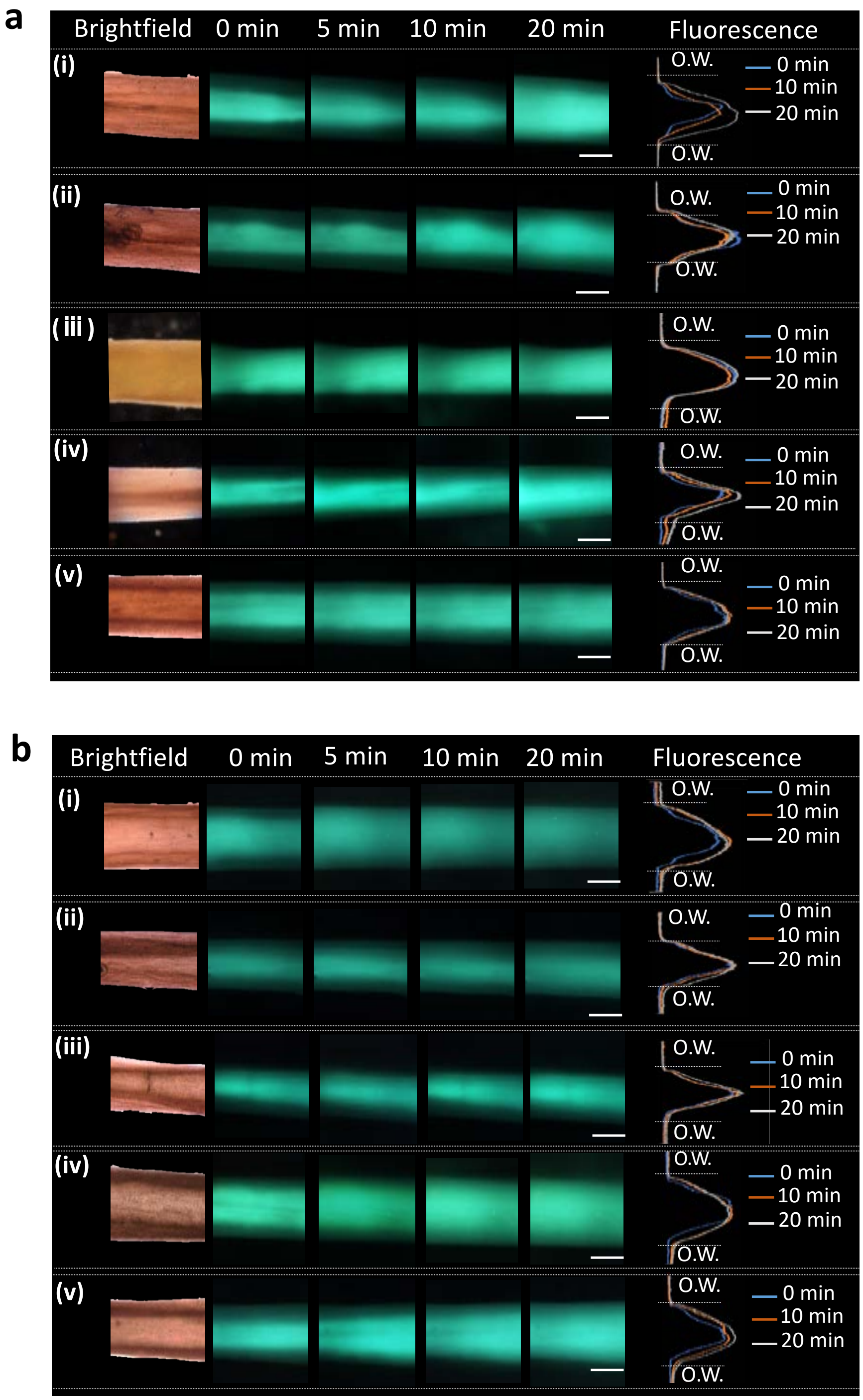

Figure S7. a,.Permeability of TEBVs to 20 µg/ml of FITC labeled 500 kD Dextran for (i) collagen tubing without cells and without prefusion; (ii) TEBVs with the cells under static culture; (iii) TEBVs with the cells and 1 week of perfusion; (iv) TEBVs after 96 h eLDL exposure; AND (v) 8 days recovery of TEBVs after eLDL treatment. **b.** Permeability of TEBVs to 10 µg/ml of FITC labeled goat IgG for (i) collagen tubing without cells and without prefusion; (ii) TEBVs with the cells under static culture. (iii) TEBVs with the cells and 1 week of perfusion; (iv) TEBVs after 96 h eLDL exposure; (v) 8 days recovery of TEBVs after eLDL treatment. (scale bar 500 µm).

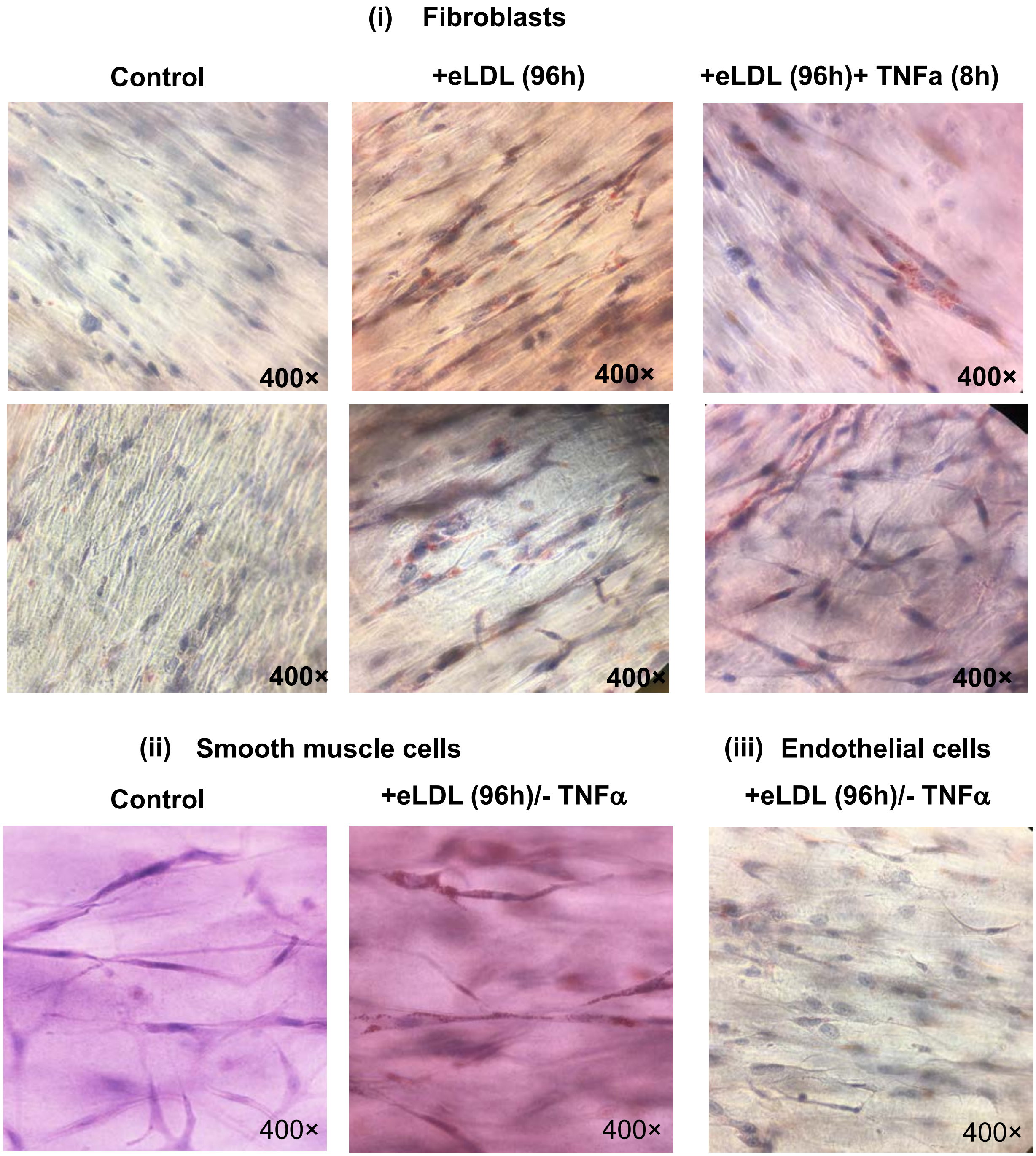

Figure S8. Oil-red staining of fibroblast, smooth muscle cells and endothelial cells in TEBVs under 96h eLDL exposure ±TNFa.

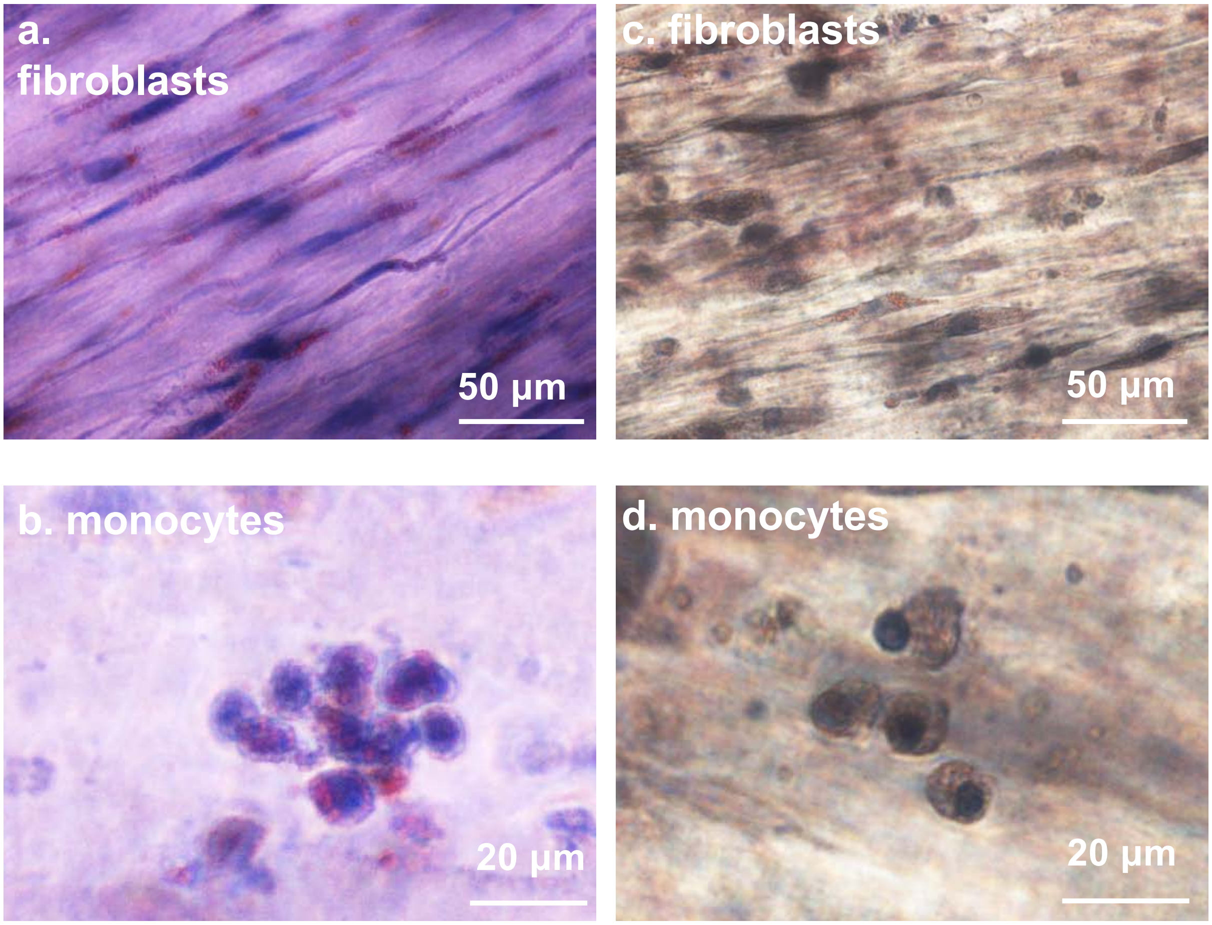

Figure S9. Images of oil red staining in TEBVs after treatment with 1 µM Lovastatin and 50 µg/mL eLDL for 96 h (scale bar: 50 µm for a and c, 20 µm for b and d).

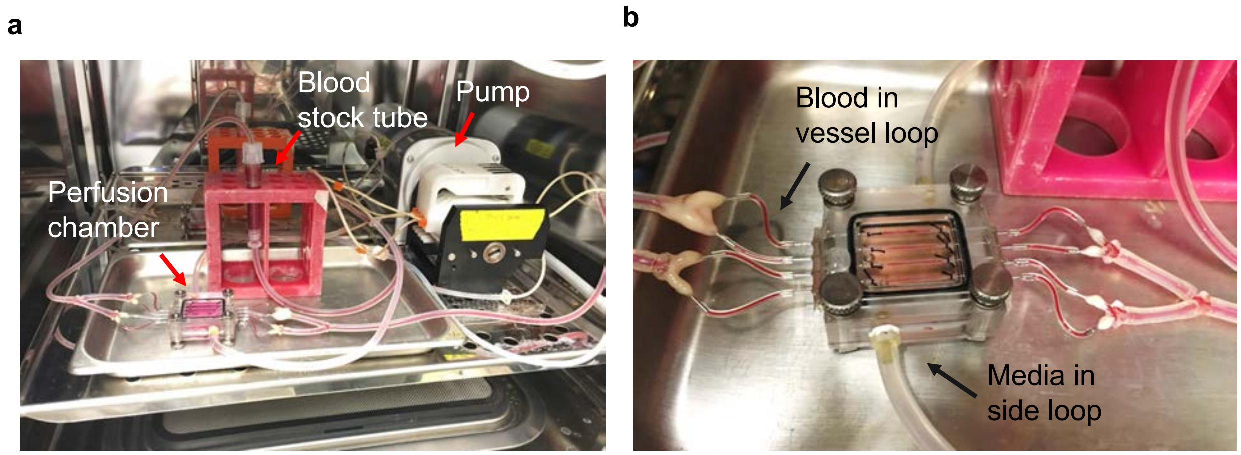

Figure S10. Images of perfusion with whole blood

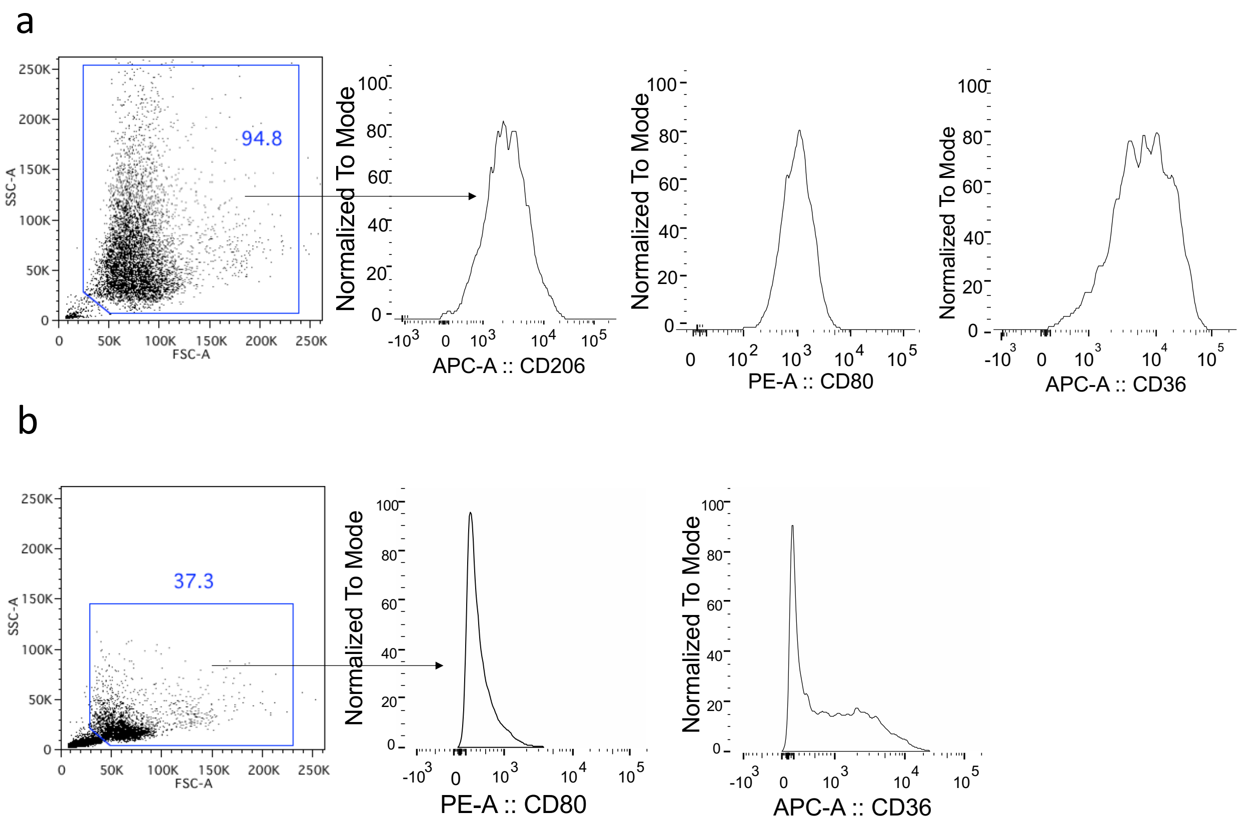

Supplementary Fig. 11 | Gating strategies for flow cytometry analysis.
11a, Representative gating strategy used to detect CD206, CD80 or CD36 expression on cultured monocytes. This gating strategy was used in Figure 7a and 7b.
11b, Representative gating strategy used to detect CD80 or CD36 expression on cultured monocytes. This gating strategy was used in Figure 8e and 8f.
