# Supplementary material for: Modeling early stage atherosclerosis in a primary human vascular microphysiological system
Source: Nat Commun. 2020 Oct 27;11:5426. doi: 10.1038/s41467-020-19197-8 (PMC7591486; doi:10.1038/s41467-020-19197-8)
Supplement: Supplementary file 3 — Description of Additional Supplementary Files [file 41467_2020_19197_MOESM3_ESM.pdf]

Description of Additional Supplementary Files

File Name: Supplementary Movie 1

Description: The video shows that the collagen TEBV does not permit fluid extravasation as shown by perfusion of a dye through the lumen.
