# Supplementary material for: Modeling early stage atherosclerosis in a primary human vascular microphysiological system
Source: Nat Commun. 2020 Oct 27;11:5426. doi: 10.1038/s41467-020-19197-8 (PMC7591486; doi:10.1038/s41467-020-19197-8)
Supplement: Supplementary file 5 — Reporting Summary [file 41467_2020_19197_MOESM5_ESM.pdf]

nature research

Corresponding author(s): George A. Truskey

Last updated by author(s): 2020-9-18

Reporting Summary

Nature Research wishes to improve the reproducibility of the work that we publish. This form provides structure for consistency and transparency in reporting. For further information on Nature Research policies, see our Editorial Policies and the Editorial Policy Checklist.

Statistics

For all statistical analyses, confirm that the following items are present in the figure legend, table legend, main text, or Methods section.

n/a	Confirmed	
☐	☒	The exact sample size (*n*) for each experimental group/condition, given as a discrete number and unit of measurement
☐	☒	A statement on whether measurements were taken from distinct samples or whether the same sample was measured repeatedly
☐	☒	The statistical test(s) used AND whether they are one- or two-sided<br>*Only common tests should be described solely by name; describe more complex techniques in the Methods section.*
☐	☒	A description of all covariates tested
☐	☒	A description of any assumptions or corrections, such as tests of normality and adjustment for multiple comparisons
☐	☒	A full description of the statistical parameters including central tendency (e.g. means) or other basic estimates (e.g. regression coefficient) AND variation (e.g. standard deviation) or associated estimates of uncertainty (e.g. confidence intervals)
☐	☒	For null hypothesis testing, the test statistic (e.g. *F*, *t*, *r*) with confidence intervals, effect sizes, degrees of freedom and *P* value noted<br>*Give P values as exact values whenever suitable.*
☒	☐	For Bayesian analysis, information on the choice of priors and Markov chain Monte Carlo settings
☒	☐	For hierarchical and complex designs, identification of the appropriate level for tests and full reporting of outcomes
☒	☐	Estimates of effect sizes (e.g. Cohen's *d*, Pearson's *r*), indicating how they were calculated

Our web collection on statistics for biologists contains articles on many of the points above.

Software and code

Policy information about availability of computer code

Data collection	1.The software "NIS-Elementa AR v3.2" was used for fluorescent microscrope<br>2.The software "ISCapture v3.6" was used for videos recording<br>3.The software "LAS AF v2.7.3.9723"was used for confocal imaging<br>4.The software "Flowjo, MAC version,v9.3.2 " was used for flow cytometry testing
Data analysis	ImageJ-Fiji 1.52i was used to analyze the fluorescence intensity and to rebuild 3D view of images, Graphpad Prism 6, OriginLab/OriginPro.v8.5 and Microsoft Excel (Office 2016 from Duke OIT) were used to perform statistical analysis. Flowjo, MAC version,v9.3.2 was used for flow cytometry analysis.

For manuscripts utilizing custom algorithms or software that are central to the research but not yet described in published literature, software must be made available to editors and reviewers. We strongly encourage code deposition in a community repository (e.g. GitHub). See the Nature Research guidelines for submitting code & software for further information.

Data

Policy information about availability of data

All manuscripts must include a data availability statement. This statement should provide the following information, where applicable:

- Accession codes, unique identifiers, or web links for publicly available datasets
- A list of figures that have associated raw data
- A description of any restrictions on data availability

The authors declare that all data supporting the findings of this study are available within the article and its Supplementary Information Files or can be requested from the authors.

Field-specific reporting

Please select the one below that is the best fit for your research. If you are not sure, read the appropriate sections before making your selection.

☒ Life sciences ☐ Behavioural & social sciences ☐ Ecological, evolutionary & environmental sciences

For a reference copy of the document with all sections, see nature.com/documents/nr-reporting-summary-flat.pdf

Life sciences study design

All studies must disclose on these points even when the disclosure is negative.

Sample size	Sample sizes were based on our previous experience with the TEBVs (Sci. Rep. 6: 21579 (2016) ; Sci. Rep. 7(1):8168 (2017)) in which we were able to demonstrate significant differences in function with 4 TEBVs per group. The variances obtain in this study were comparable to the prior work.
Data exclusions	No data were excluded from the analysis except when the TEBVs were obviously or damaged broken during perfusion.
Replication	For nitric oxide (NO) testing, each group tested consisted of 2 independent experiments with 4 TEBVs in each . The other results were quantified using 2-7 independent TEBVs. The flow cytometry were tested based on 3 independent wells. Sample size was chosen based on the common practice in the field. All attempts at replication were successful.
Randomization	Randomization was not performed since all cell sources were from healthy donors and cell properties was not a goal of the study. Further, cells were not chosen in advance based on prior knowledge of responsiveness to treatments.
Blinding	Blinding was not done. After preparation of TEBVs, samples were exposed to one of the conditions reported in the figure and treatments were known to the authors.

Reporting for specific materials, systems and methods

We require information from authors about some types of materials, experimental systems and methods used in many studies. Here, indicate whether each material, system or method listed is relevant to your study. If you are not sure if a list item applies to your research, read the appropriate section before selecting a response.

Materials & experimental systems

n/a	Involved in the study	
☐	☒	Antibodies
☐	☒	Eukaryotic cell lines
☒	☐	Palaeontology and archaeology
☒	☐	Animals and other organisms
☐	☒	Human research participants
☒	☐	Clinical data
☒	☐	Dual use research of concern

Methods

n/a	Involved in the study	
☒	☐	ChIP-seq
☐	☒	Flow cytometry
☒	☐	MRI-based neuroimaging

Antibodies

Antibodies used

Purified anti-humanvCD80, Biolegend, Clone 2D10, Cat305202, Lot B258948
Purified Mouse anti-human CD31, BD Pharmingen, Cat 550389, Lot 7129558
VCAM-1, mouse monoclonal IgG, Santa Cruz Biotech., sc-13160, LotG3014
ICAM, mouse monoclonal IgG1, Santa Cruz Biotech., sc-107, Lot H1417
E-Selectin, mouse monoclonal IgG2a, Santa Cruz Biotech., sc-137054, Lot D1619
Rabbit pAb to alpha smooth muscle actin, abcam, ab5694, Lot GR3183259-31
APC Mouse Anti-human CD206, BD Pharmingen, Cat 550889, Lot 8005527
Pacific Blue anti-human CD14, Biolegend, clone 63D3, Cat 367121, Lot B271628
APC anti-human CD36, Biolegend, clone 5-271, Cat 336207, Lot B257709
PE anti-human CD80, Biolegend, clone 2D10, Cat 305207, Lot B266816

Validation

Antibodies were validated by the manufacturer using flow cytometry, Western blotting, or immunostaining as follows:

Purified anti-human CD80, Biolegend, Clone 2D10, Cat305202, Lot B258948: Host Species-Mouse; Application: FC - Quality tested, IP, WB, IHC-F - Reported in the literature; This product lot has passed BioLegend's QC testing and is certified for use. For details on QC testing view our page at biolegend.com/en-us/quality-control.

Purified Mouse anti-human CD31, BD Pharmingen, Cat 550389, Lot 7129558: Isotype: Mouse IgG1, κ; Reactivity: Human (QC Testing) Rhesus, Cynomolgus, Baboon (Tested in Development); Application: Flow cytometry (Routinely Tested) , Immunohistochemistry-

frozen, Immunohistochemistry-zinc-fixed (Tested During Development) ,Immunohistochemistry-formalin (antigen retrieval required) (Not Recommended);

VCAM-1, mouse monoclonal IgG, Santa Cruz Biotech., sc-13160, LotG3014: Species: VCAM-1 (E-10) is a mouse monoclonal antibody raised against amino acids, 25-300 of VCAM-1 of human origin; Application: VCAM-1 (E-10) is recommended for detection of VCAM-1 of mouse, rat and human origin by Western Blotting (starting dilution 1:100, dilution range 1:100-1:1,000), immunoprecipitation [1-2 µg per 100-500 µg of total protein (1 ml of cell lysate)], immunofluorescence (starting dilution 1:50, dilution range 1:50-1:500), immunohistochemistry (including paraffin-embedded sections) (starting dilution 1:50, dilution range 1:50-1:500), flow cytometry (1 µg per 1 x 106 cells) and solid phase ELISA (starting dilution 1:30, dilution range 1:30-1:3000)

ICAM-1, mouse monoclonal IgG1, Santa Cruz Biotech., sc-107, Lot H1417:Species: ICAM-1 (15.2) is a mouse monoclonal antibody raised against an ICAM-1 positive cell preparation; Application:ICAM-1 (15.2) is recommended for detection of ICAM-1 of mouse, rat and human origin by Western Blotting (starting dilution 1:200, dilution range 1:100-1:1000), immunoprecipitation [1-2 µg per 100-500 µg of total protein (1 ml of cell lysate)], immunofluorescence (starting dilution 1:50, dilution range 1:50-1:500), immunohistochemistry (including paraffin-embedded sections) (starting dilution 1:50, dilution range 1:50-1:500) and flow cytometry (1 µg per 1 x 106 cells).

E-Selectin, mouse monoclonal IgG2a, Santa Cruz Biotech., sc-137054, Lot D1619:Species: E-Selectin (D-7) is a mouse monoclonal antibody raised against amino acids 311-610 mapping at the C-terminus of E-Selectin of human origin; Application:E-Selectin (D-7) is recommended for detection of E-Selectin of mouse, rat and human origin by Western Blotting (starting dilution 1:100, dilution range 1:100-1:1000), immunoprecipitation [1-2 µg per 100-500 µg of total protein (1 ml of cell lysate)], immunofluorescence (starting dilution 1:50, dilution range 1:50-1:500), immunohistochemistry (including paraffin-embedded sections) (starting dilution 1:50, dilution range 1:50-1:500) and solid phase ELISA (starting dilution 1:30, dilution range 1:30-1:3000).

MHC11,Rabbit monoclonal [EPR5336(B)] to smooth muscle Myosin heavy chain 11, Abcam, ab133567: Host species-Rabbit; Tested applications: Suitable for: Flow Cyt, WB, IHC-P, ICC/IFmore details; Species reactivity: Reacts with Human; Positive control :IHC: Human ovary and prostate tissues; WB: Human bladder, artery and testis tissue lysates; Flow Cyt: 293 cells; ICC: Human smooth muscle and A673 cells.

Rabbit pAb to alpha smooth muscle actin, abcam, ab5694, Lot GR3183259-31: Host species-Rabbit; Tested applications: Suitable for: WB, IHC-Pmore details; Species reactivity: Reacts with: Mouse, Chicken, Cow, Dog, Human, Pig; Purity: Immunogen affinity purified.

APC Mouse Anti-human CD206, BD Pharmingen, Cat 550889, Lot 8005527: IsotypeMouse IgG1, κ; Reactivity: Human (QC Testing); Application: Flow cytometry (Routinely Tested)

Pacific Blue anti-human CD14, Biolegend, clone 63D3, Cat 367121, Lot B271628:Host Species-Mouse; Application: FC - Quality tested; This product lot has passed BioLegend's QC testing and is certified for use. For details on QC testing view our page at biolegend.com/en-us/quality-control.

APC anti-human CD36, Biolegend, clone 5-271, Cat 336207, Lot B257709:Host Species-Mouse; Application: FC - Quality tested; This product lot has passed BioLegend's QC testing and is certified for use. For details on QC testing view our page at biolegend.com/en-us/quality-control.

PE anti-human CD80, Biolegend, clone 2D10, Cat 305207, Lot B266816:Host Species-Mouse; Application: FC - Quality tested; This product lot has passed BioLegend's QC testing and is certified for use. For details on QC testing view our page at biolegend.com/en-us/quality-control.

APC Mouse IgG2a, Biolegend, clone MOPC-173, Cat 981906: Application: suggested for Flow Cytometry; This product has passed BioLegend's QC testing and is certified for use. For details on QC testing view our page at biolegend.com/en-us/quality-control.

PE Mouse IgG1, Biolegend, clone MOPC-21, Cat 981804:Application: suggested for Flow Cytometry; This product has passed BioLegend's QC testing and is certified for use. For details on QC testing view our page at biolegend.com/en-us/quality-control.

Pacific Blue™ Mouse IgG1, Biolegend, clone MOPC-21, Cat 981812:Application: suggested for Flow Cytometry; This product has passed BioLegend's QC testing and is certified for use. For details on QC testing view our page at biolegend.com/en-us/quality-control.

Eukaryotic cell lines

Policy information about cell lines

Cell line source(s)	Primary human endothelial colony forming cells (ECFCs) were isolated and expanded from umbilical cord blood in our lab.<br>Primary human neonatal dermal fibroblasts (hNDF) were purchased from Clonetics.<br>Primary human Coronary Artery Smooth Muscle (hCASMCs) were purchased from Cell Applications, Inc. (San Diego, CA)<br>Primary human monocytes were isolated from whole blood with Magnetic Assisted Cell Sorting in our lab.<br>The human monocytic cell line U937 was purchased from Sigma.
Authentication	All primary human ECFCs and monocytes isolated from blood were performed using a protocol approved by the Duke University Institutional Review Board (IRB). Their purity was assessed by flow cytometry or immunofluorescent staining as described in the paper. The authentication of purchased cell lines was performed by the supplier and we also verified that the U937 cells were CD14 positive and CD80 negative. For hCASMCs the manufacturer verified that the cells were positive for smooth muscle cell actin and smooth muscle myosin heavy chain 11. The manufacturer characterized hNDF by morphological observation throughout serial passage.

Mycoplasma contamination	The hNDF, hCASMCs and U937 cells were tested by the manufacturer and are negative for mycoplasma.<br>Primary human endothelial colony forming cells (ECFCs) and primary human monocytes were not tested for mycoplasma contamination.
Commonly misidentified lines<br>(See ICLAC register)	No misidentified lines were used.

Human research participants

Policy information about studies involving human research participants

Population characteristics	Umibilical cord blood was Carolina Cord Blood Bank after delivery of newborn. No idnetifying information was provided to the investigators.
Recruitment	Samples were obtained from the Carolina Cord Blood Bank within 48 h of collection. All identifiers were removed.
Ethics oversight	The Duke University Institutional Review Board determined that the protocol meets the definition of research not involving human subjects as described in 45 CFR 46.102(f), 21 CFR 56.102(e) and 21 CFR 812.3(p) and satisfies the Privacy Rule as described in 45CFR164.514.

Note that full information on the approval of the study protocol must also be provided in the manuscript.

Flow Cytometry

Plots

Confirm that:

☒ The axis labels state the marker and fluorochrome used (e.g. CD4-FITC).

☒ The axis scales are clearly visible. Include numbers along axes only for bottom left plot of group (a 'group' is an analysis of identical markers).

☒ All plots are contour plots with outliers or pseudocolor plots.

☒ A numerical value for number of cells or percentage (with statistics) is provided.

Methodology

Sample preparation	Primary human monocytes were isolated from whole blood with Magnetic Assisted Cell Sorting (MACS, CD14+Beads, Miltenyi Biotec). The cells were more than 90% CD 14+ after isolation. The treated samples were incubated with antibodies for 15min on ice for staining.
Instrument	BD Biosciences, FACSCanto II
Software	Flowjo, MAC version,v9.3.2
Cell population abundance	The purity of sorted monocyte was >90%, determined by flow-cytometry. ECFC purity was 95% as judged by flow cytometry.
Gating strategy	Preliminary FSC/SSC gating was used to gate on monocyte/macrophage population.

☐ Tick this box to confirm that a figure exemplifying the gating strategy is provided in the Supplementary Information.
